# PF2401-SF, Standardized Fraction of *Salvia miltiorrhiza*, Induces Apoptosis of Activated Hepatic Stellate Cells *in Vitro* and *in Vivo*

**DOI:** 10.3390/molecules18022122

**Published:** 2013-02-06

**Authors:** Daya Ram Parajuli, Eun-Jeon Park, Xian-Hua Che, Wen-Yi Jiang, Youn-Chul Kim, Dong Hwan Sohn, Sung Hee Lee

**Affiliations:** Department of Pharmacy, Institute of Pharmaceutical Research and Development, Wonkwang University, Iksan, Jeonbuk 570-749, Korea; E-Mails: dayaramparajui@gmail.com (D.R.P.); ejp70@wku.ac.kr (E.-J.P.); xianhua128@hotmail.com (X.-H.C.); roy_fido@naver.com (W.-Y.J.); yckim@wku.ac.kr (Y.-C.K.)

**Keywords:** *Salvia miltiorrhiza*, hepatic stellate cells, caspases, apoptosis

## Abstract

During the course of our attempts to develop a potential herbal medicine, we had previously prepared PF2401-SF, a standardized fraction of *S. miltiorrhiza*, and reported its hepatoprotective activity *in vitro* as well as *in vivo*. Since apoptosis of activated hepatic stellate cells (HSCs) is a well-accepted anti-fibrotic strategy, in this study, we investigated the direct effect of PF2401-SF on t-HSC/Cl-6 cells *in vitro* and on CCl_4_-induced liver injury *in vivo*. We evaluated the activation and cleavage of hallmarkers of apoptosis, namely, caspase 3, 8, 9 and PARP. Upregulation of the pro-apoptotic Bax protein and downregulation of the anti-apoptotic Bcl2 protein were also analyzed. Furthermore, in the PF2401-SF treated rats, apoptosis induction of activated HSCs was demonstrated by reduced distribution of α-SMA-positive cells and the presence of high number of TUNEL-positive cells *in vivo*. Our data suggest that PF2401-SF can mediate HSCs apoptosis induction, and may be a potential herbal medicine for the treatment of liver fibrosis.

## 1. Introduction

Liver fibrosis is a wound-healing response to chronic liver injuries associated with diverse etiological factors [1,2]. The progressive pathological process underlying hepatic fibrosis is the activation of quiescent hepatic stellate cells into proliferative and contractile myofibroblast-like cells with abnormal and exaggerated deposition of extracellular matrix [3,4]. Hepatic stellate cells (HSCs) are the specific cellular targets for anti-fibrotic therapy in chronic liver diseases of any etiology [5,6]. Termination of the proliferation of activated HSCs by apoptosis has been proposed as an exciting therapy for patients with chronic liver injury and fibrosis [7,8]. Despite some promising results, no effective and targeted anti-fibrotic drugs have yet been licensed, which is a clear indicator of the need for studying liver diseases and the challenges inherent in these studies.

*Salvia miltiorrhiza* Bunge (Labiatae) has been widely used for the treatment of various liver diseases in traditional Chinese medicine [9,10] and the anti-fibrotic effects of *S. miltiorrhiza* have also been reported in different experimental models [11,12,13,14]. Despite the popularity of *S. miltiorrhiza* for treatment of liver diseases, scientific data associated with its quality standardization were distinctly lacking. We had previously prepared a standardized and purified fraction of *S. miltiorrhiza*, PF2401-SF, and demonstrated that PF2401-SF and its major components protect hepatocytes *in vitro* and *in vivo* [15,16]. We strongly believe that the PF2401-SF, needs further scientific evaluation to develop into a potential herbal medicine for liver diseases because, an ideal anti-fibrotic agent with good clinical application should induce apoptosis of HSCs with no harmful effects on hepatocytes and liver functions [17]. Therefore, the objective of this study was to evaluate the direct effect of PF2401-SF on activated HSCs. We investigated the apoptotic effect of PF2401-SF in t-HSC/Cl-6 cells *in vitro* and CCl_4_-in duced liver injury *in vivo*.

## 2. Results

### 2.1. Effect of PF2401-SF on Cell Viability

**Figure 1:**
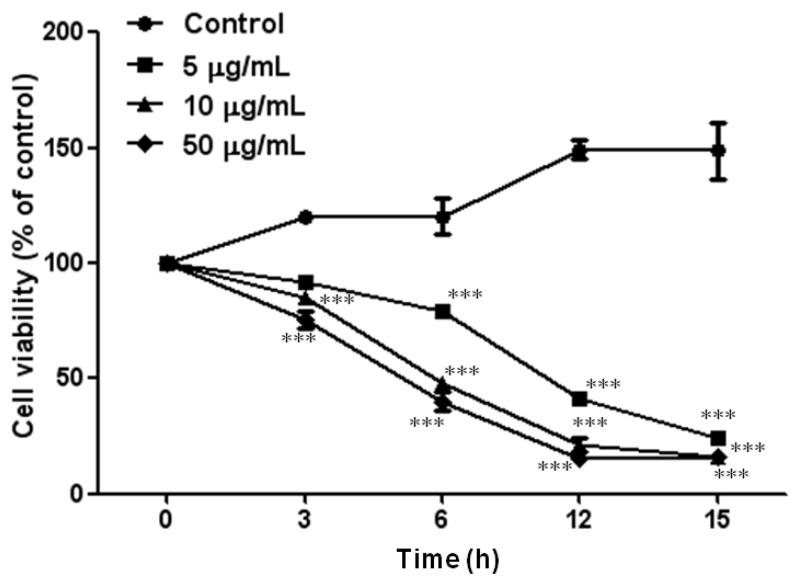
t-HSC/Cl-6 cells were treated with 0–50 µg/mL of PF2401-SF for 0–15 h. Results are expressed as percentage control of cell viability at the corresponding point measured by MTT assay. Data are presented as mean ± S.D. for each point. * *p* < 0.05, ** *p* < 0.01, *** *p* < 0.001 compared to control cells treated with DMSO.

To examine the cytotoxic effect of PF2401-SF (0, 5, 10, and 50 µg/mL) on t-HSC/Cl-6 cells, an MTT assay was performed. The reduction of cell viability was better in higher doses compared to small dose. At 50 µg/mL, cell viability was reduced to 75.5%, 40%, 15.3% and 16.3% following exposure for 3, 6, 12 and 15 h, respectively (Figure 1). Therefore, we chose 20 µg/mL as an optimum dose for the further experiments.

### 2.2. Effect of PF2401-SF on Apoptosis Induction *in Vitro*

**Figure 2:**
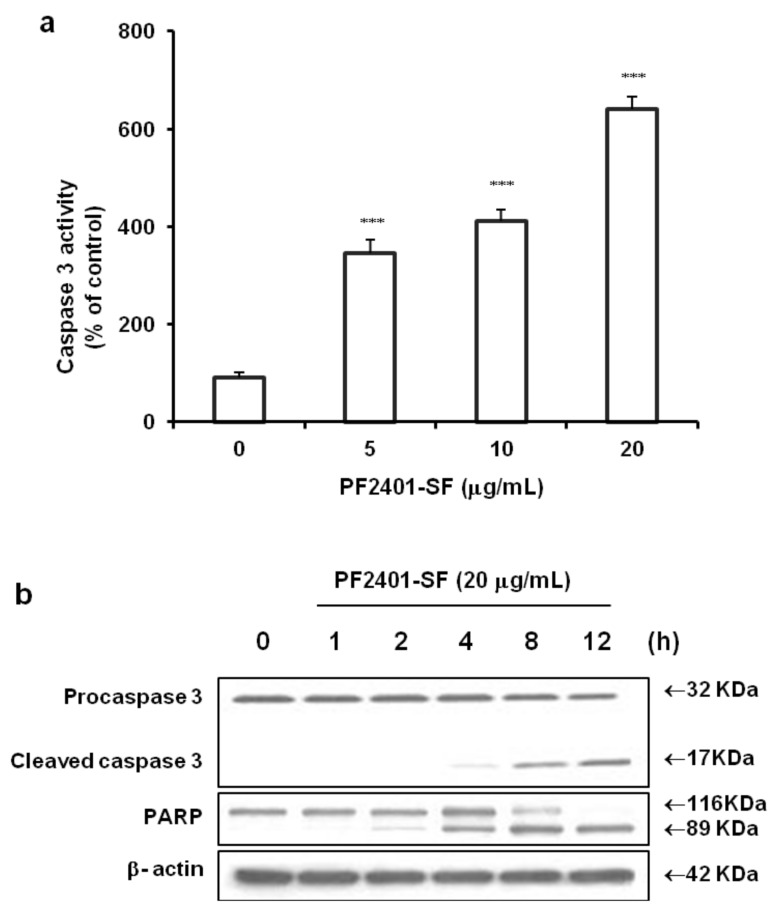
Apoptosis induction in t-HSC/Cl-6 cells treated with PF2401-SF for 12 h at different concentrations. Caspase 3 was measured by colorimetric assay using DEVD-pNA substrate. A strong effect was observed at 20 µg/mL (**a**). Data are presented as mean ± S.D. for each point. *** *p* < 0.001 compared to control. Western blot analysis revealed time dependent cleavage of caspase 3 and PARP indicating apoptosis induction in t-HSC/Cl-6 cells exposed to PF2401-SF for 12 h (**b**).

**Figure 3:**
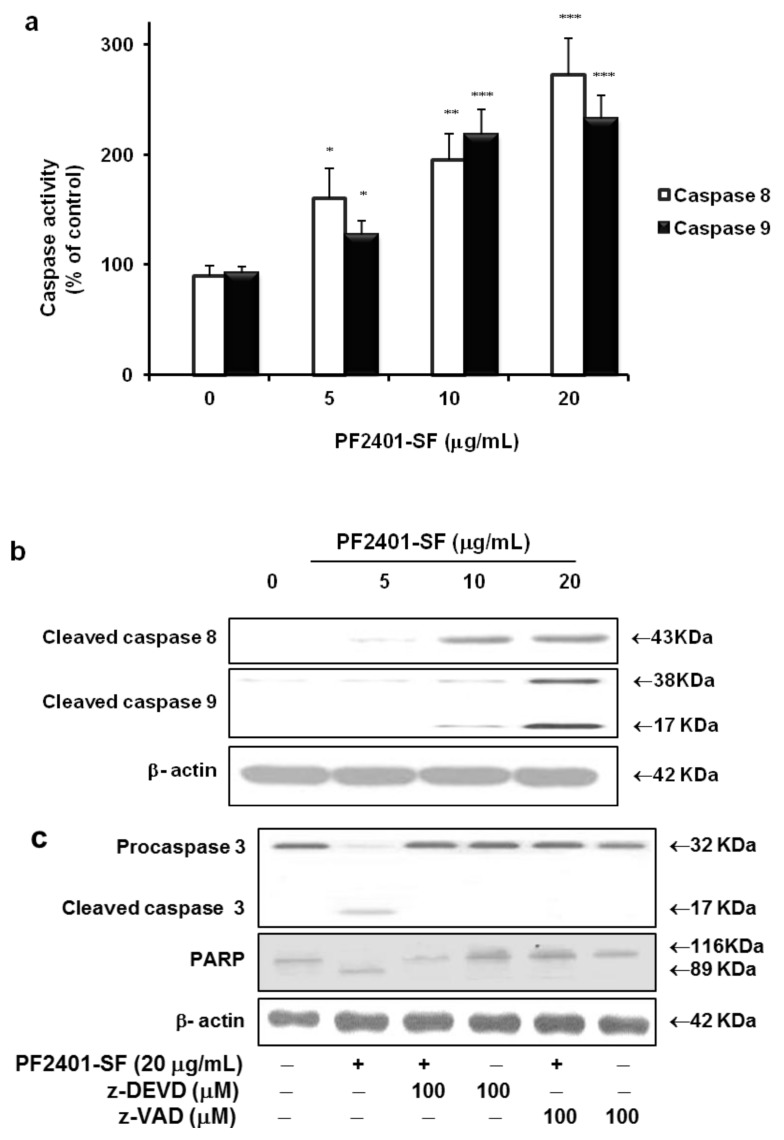
Colorimetric determination using IETD-pNA and LEHD-pNA substrates for caspase 8 and 9 exposed with PF2401-SF for 12 h resulted in dose-dependent activation of both caspases (**a**). Data are presented as mean ± S.D. for each point. * *p* < 0.05, ** *p* < 0.01, *** *p* < 0.001 compared with the control. Similarly, dose-dependent cleavage of caspase 8 and 9 was observed from western blot analysis (**b**). Cells were pretreated for 2 h with z-DEVD-fmk (caspase 3 inhibitor) and Z-VAD-fmk (pan-caspase inhibitor) followed by treatment with PF2401-SF for 12 h. PF2401-SF induced cleavage of caspase 3 and PARP was inhibited by pretreatment with caspase inhibitors (**c**).

The fate of PF2401-SF-induced cytotoxicity might involve apoptosis, and family of caspase enzymes gets activated during apoptotic signaling events. Hence, we used a colorimetric assay with DEVD-pNA substrate to measure the protease activity of caspase 3. PF2401-SF treatment 20 µg/mL for 12 h significantly increased caspase 3 activity in dose-dependent manner (Figure 2a). Active form of biochemical hallmarkers of apoptosis, *i.e.*, caspase 3 and PARP were observed in a time-dependent manner in western blot analysis (Figure 2b). The upstream caspase 8 and 9 were also activated (Figure 3a) and underwent cleavage (Figure 3b), suggesting that PF2401-SF-induced apoptosis in t-HSC/Cl-6 cells.

To confirm the role of caspases in PF2401-SF mediated apoptosis, t-HSC/Cl-6 cells were pretreated for 2 h with specific cell-permeable caspase inhibitors. Then apoptosis was induced in these cells by treatment with PF2401-SF (20 µg/mL) for 12 h. z-DEVD-fmk (caspase 3 inhibitor) and Z-VAD-fmk (pan-caspase inhibitor) significantly inhibited PF2401-SF induced apoptosis and the cleavage of caspase 3 and PARP (Figure 3c). This data demonstrate that the PF2401-SF-mediated apoptosis in t-HSC/Cl-6 cells involve a caspase-dependent pathway.

### 2.3. Effect of PF2401-SF on Pro-Apoptotic and Anti-Apoptotic Proteins

**Figure 4:**
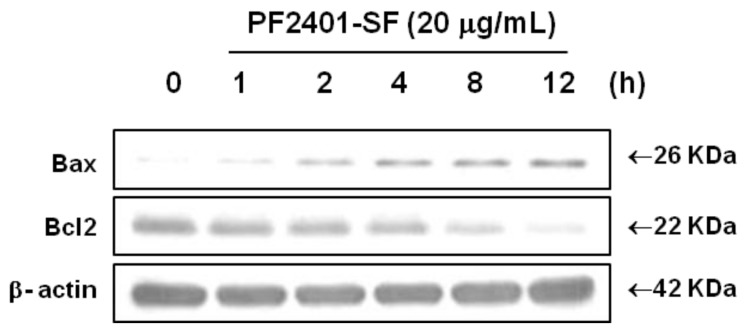
Effect of PF2401-SF on pro-apoptotic and anti-apoptotic proteins. Time-dependent downregulation of Bcl2 and upregulation of Bax was found in t-HSC/Cl-6 cells treated with 20 µg/mL PF2401-SF and analyzed by western blot. Regulation of these mitochondrial proteins correlates with apoptosis induction *in vitro*.

To further elucidate the signal transduction pathway involving the members of the caspase families in PF2401-SF induced apoptosis, we investigated changes in the levels of proteins belonging to the mitochondrial apoptotic pathway. In PF2401-SF treated cells, the upregulation of pro-apoptotic Bax and downregulation of anti-apoptotic Bcl2 started from 1 h in time-dependent manner (Figure 4). This result also suggests the induction of apoptosis in t-HSC/Cl-6 cells *in vitro*.

### 2.4. Effect of PF2401-SF on Apoptosis Induction *in Vivo*

**Figure 5:**
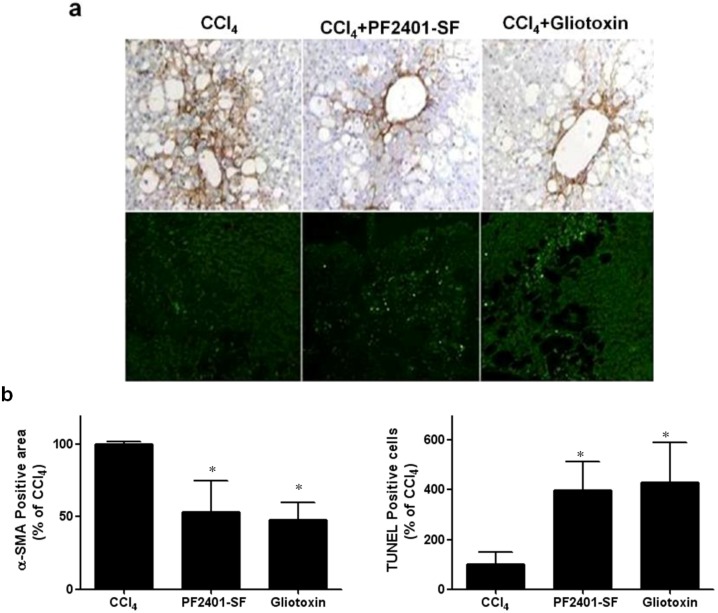
Effect of PF2401-SF on expression of α-SMA in CCl_4_ intoxicated rat liver (**a**, upper panel). The expression of α-SMA, a typical marker of activated HSCs in the liver was detected by immunohistochemical staining. Rats were given repeated injection of CCl_4_ (0.75 mL/kg, p.o., diluted in corn oil) every 3 days for 13 days. Either PF2401-SF (50 mg/kg, p.o., 1 h after CCl_4_ administration on day 13 and 14) or reference drug gliotoxin (3 mg/kg, i.p., day 14) were also administered, and liver tissues were obtained on day 15. TUNEL assay for determination of apoptotic cells (**a**, lower panel). The apoptotic cells marked by green fluorescence. Densitometry data showed the statistically significant (* *p* < 0.05) difference for the PF2401-SF induced dowregulation of α-SMA-positive cells and increased number of TUNEL-positive cells comparing to CCl_4_ group (**b**). Representative photographs (n = 5 rats) are shown (original magnification ×100).

To investigate whether PF2401-SF induces apoptosis *in vivo*, a subacute CCl_4_ rat model was developed. CCl_4_ administration activates quiescent HSCs into α-SMA-positive and proliferating myofibroblast-like cells [18]. There was no α-SMA-positive staining in the control group (data not shown). The expression of α-SMA protein in PF2401-SF- treated group was much lower than that in the CCl_4_ group (Figure 5a, upper panel). The effect of PF2401-SF on reduction of α-SMA-positive cells was highly comparable with that of gliotoxin, a well-studied HSCs apoptotic agent [19]. Apoptosis is suggested to be the process underlying elimination of activated HSCs in liver injury. We further examined whether the reduction in α-SMA-positive cells was because of apoptosis. The number of apoptotic cells in the control group (data not shown) and CCl_4_ group were extremely low. However, many apoptotic cells were observed in the PF2401-SF and Gliotoxin-treated groups (Figure 5a, lower panel). Densitometry data also showed the statistically significant difference for the PF2401-SF induced dowregulation of α-SMA-positive cells and increased number of TUNEL-positive cells comparing to CCl_4_ group (Figure 5b). Accumulating evidence possibly explains the reduction in activated HSCs through increasing apoptosis *in vivo*.

## 3. Discussion

Liver fibrosis and its end-stage disease cirrhosis are a major health burden affecting millions of people worldwide [20,21]. Herbal medicines are an example of the perfection that nature has given us to survive. Herbal medicines have long history of application as hepatoprotective and anti-fibrotic agents [22,23]. Research on PF2401-SF is a part of on-going project in our laboratory to develop a potential herbal medicine and rationalize its use in liver fibrosis. We previously reported that PF2401-SF was hepatoprotective at 50–200 mg/kg per day in acute liver injury as well as 25–100 mg/kg per day in subacute liver injury [16]. It is well accepted that liver fibrosis can be reversed through the apoptosis of activated HSCs [8,24]. Therefore, in this study, we investigated the apoptotic effect of PF2401-SF in t-HSC/Cl-6 cells *in vitro* and CCl_4_-induced liver injury *in vivo.*

After measurement of cytotoxic effect of PF2401-SF in t-HSC/Cl-6 cells using the MTT assay, we further attempted to elucidate the signaling pathway of apoptosis. Caspases are synthesized as catalytically inactive pro-enzymes and need to be activated by proteolytic cleavage [25]. Strong activation and cleavage of all three caspases were observed in our study in consistent with the reports of recent study, cleavage of caspase 3, 8, 9 and PARP are the hallmark of apoptosis [26]. We found that PF2401-SF-induced apoptosis was blocked by z-DEVD-fmk, a specific caspase 3 inhibitor, and z-VAD-fmk, a pan-caspase inhibitor indicating the role of caspases in apoptosis induction. Our data demonstrated the co-existence of the two pathways mediated by caspase 8-dependent death receptor pathway and caspase 9- and 3-mediated mitochondrial pathway [27]. Caspase 3 is one of the downstream effector caspases and is a key protease responsible for the cleavage and inactivation of PARP [28]. Time and dose-dependent cleavage of PARP was observed in PF2401-SF treated t-HSC/Cl-6 cells, correlating with the findings of a previous report that cleavage of PARP contribute to ongoing apoptosis [29].

The mitochondrial pathway of apoptosis is regulated by members of the Bcl-2 family of proteins [30]. The intrinsic pathway of apoptosis is mediated through mitochondria and results in efflux of cytochrome c and other pro-apoptotic proteins into the cytosol via mitochondrial outer membrane permeabilization [31]. Once in the cytosol, these proteins stimulate caspase activation which in turn causes cellular demise [32]. In our study, the pro-apoptotic protein Bax was upregulated whereas the anti-apoptotic protein Bcl2 was downregulated in a time-dependent manner upon treatment with PF2401-SF, further potentiating the induction of apoptosis in t-HSC/Cl-6 cells. These data suggest a mitochondrial signaling pathway for induction of apoptosis.

Apoptosis has been investigated extensively as a vital mechanism to minimize the number of activated HSCs during the resolution phase of liver injury. Experimental models and clinical studies have demonstrated the correlation between the apoptosis of stellate cells and a regression of fibrosis [33]. We determined whether PF2401-SF induces apoptosis of activated HSCs in rat model of subacute CCl_4_ intoxication. Our study revealed the reduction in the distribution of α-SMA-positive cells, a typical marker of activated HSCs during the fibrotic process [34]. Involvement of apoptosis to reduce α-SMA-positive cells was further elucidated by increase number of TUNEL-positive cells around the fibrotic area. These data suggest that PF2401-SF effectively induces the apoptosis of activated HSCs *in vivo* at a dose of 50 mg/kg, a range we showed in our previous study to be hepatoprotective [16].

## 4. Experimental

### 4.1. Preparation of PF2401-SF

A standardized and purified extract PF2401-SF was prepared from the dried, pulverized roots of *S. miltiorrhiza* as reported previously [15]. The roots of *S. miltiorrhiza* were purchased from the University Oriental Herbal Drugstore, Iksan, Korea, and were identified by Dr. Kyu-Kwan Jang, Botanical Gardens, and Wonkwang University, Korea. A voucher specimen (No. WP05-87) was deposited at the Herbarium of the College of Pharmacy, Wonkwang University.

Dried and pulverized roots of *S. miltiorrhiza* (2 kg) were soaked in 1.6 L distilled water for 12 h at room temperature, extracted with hot ethanol for 2 h, and filtered with filter paper. The filtrate was evaporated *in vacuum* to produce an ethanol extract of 277 g. The ethanol extract was suspended in distilled water (500 mL), followed by filtration. The residue derived from the filtration was dissolved in hot ethanol (95.6% at 78 °C). and filtered again. The filtrate was then evaporated in vacuum to obtain a standardized fraction of *S. miltiorrhiza* (PF2401-SF, 20 g, 1.0 w/w %).

### 4.2. Chemical Reagents

Monoclonal anti-β-actin antibody, monoclonal anti-α-smooth muscle actin (α-SMA, clone 1 A4) and 3-(4,5-dimethylthiazol-1-yl)-2,5-diphenyltetrazolium bromide (MTT) were purchased from Sigma Chemical Co. (St. Louis, MO, USA). Caspases 3 was purchased from Santa Cruz, Inc, (Santa Cruz, CA, USA). Cleaved caspase 8 and 9, Bcl2, Bax and poly(ADP-ribose) polymerase (PARP) were purchased from Cell Signaling Technology (Beverly, MA, USA). Horseradish-peroxidase (HRP)—conjugated goat anti-rabbit IgG, goat anti-mouse IgG secondary antibody, Z-VAD-fmk (pan-caspase inhibitor), and z-DEVD-fmk (caspase 3 inhibitor) were bought from R&D Systems, Inc. (Minneapolis, MN, USA). All other chemicals used were commercially available reagents of analytical quality.

### 4.3. Cell Culture

Hepatic stellate cell line was transformed using *Simian virus* 40 to obtain sufficient quantities of HSCs for studying subcultures. The hepatic stellate cell line made was named t-HSC/Cl-6, which retains the features of activated stellate cells [35]. Cells were cultured in Williams medium E (WME) supplemented with 10% fetal bovine serum (FBS), 100 units/mL penicillin G, and 100 mg/mL streptomycin at 37 °C under 5% CO_2_/95% O_2_.

### 4.4. Cell Viability Measurement

Cell viability was determined by MTT assay. Cells were seeded in 24-well plates (5 × 10^5^ cells/well). After incubation for 24 h, cells were treated with different concentrations of PF2401-SF and MTT assay performed in various time intervals (Figure 1). MTT solution (250 µL, 0.5 mg/mL made with 10% FBS culture medium) was added to every well followed by incubation of cells for 2 h. Medium containing MTT was aspirated off, and 200 µL of dimethyl sulfoxide (DMSO) was added to each well to solubilize formazan crystals formed. From each well, 100 µL of blue colored solution was transferred to 96-well plate and keep for 5 min over homogenizer for mixing followed by measurement of absorbance at 540 nm using a microplate reader (Molecular Devices Co., Sunnyvale, CA, USA).

### 4.5. Caspase 3, 8 and 9 Colorimetric Assay

Colorimetric assay kits (R&D Systems, Inc.) were used to measure the enzyme activities of different caspases according to the manufacturer’s instructions. Cells that are suspected to or have been induced to undergo apoptosis were first lysed to collect their intracellular contents. The cell lysate can then be tested for protease activity by the addition of a caspase-specific peptide that is conjugated to the color reporter molecule p-nitroaniline (pNA). The cleavage of the peptide by the caspase releases the chromophore pNA, which can be quantified spectrophotometrically at a wavelength of 405 nm by using a microplate reader (Molecular Devices Co.). Protein concentration in t-HSC/Cl-6 cell lysate was determined with a protein assay kit (Bio-Rad Laboratories, Hercules, CA, USA).

### 4.6. Western Blot Analysis

Cells treated with PF2401-SF were collected by scrapping and centrifuged at 3,000 rpm at 4 °C for 5 min. Supernatant removed and cell pellets resuspended with phosphate buffer saline (PBS) followed by solubilization in lysis buffer (10 mM Tris, pH 8.0, 120 mM NaCl, 0.5% Nonidet P40, 1 mM EDTA, 0.1 mM PMSF, 1 mM dithiothreitol, and 1 μg/mL aprotinin) on ice for 30 min. The lysate was centrifuged at 14,000 rpm at 4 °C for 25 min to remove insoluble materials. Supernatant transferred to new eppendorf tube and protein determination was performed with the DC protein assay kit (Bio-Rad). Samples were boiled at 100 °C for 5 min and 40–50 μg of protein/lane was loaded to 10% or 12% SDS-polyacrylamide gel electrophoresis, so that the gel gets electrotransfered onto nitrocellulose membranes. Ponceau-S staining was performed to assure equal protein loading and correct blotting. After blocking with 5% nonfat milk in PBS for 1 h at room temperature, the blots were probed overnight at 4 °C with the indicated primary antibodies including; PARP (1:1,000), caspase 3, 8, 9 (1:1,000), Bcl-2 (1:1,000) and Bax (1:1,000). Individual level of target protein expression was normalized to β-actin. Subsequently, membranes incubated with either horseradish peroxidase—conjugated goat anti-rabbit IgG (1:2,000) or goat anti-mouse IgG (1:1,500) secondary antibodies. Finally, membrane was washed with PBS containing 0.1% Tween-20 (PBST) and peroxidase activity was detected using a chemiluminescent reagent (Lumi-GLO; Kirkegaard & Perry Laboratories, Inc., Gaithersburg, MD, USA) according to the manufacturer’s instructions.

### 4.7. Animals

Male Sprague-Dawley rats (200–220 g) were supplied by Dae Han Laboratory Animal Research and Co. (Chungbuk, Korea), and fed with a normal standard chowdiet (Jae IL Chow, Chungbuk, Korea) and tap water *ad libitum*. Animal experiments were performed under the “Guiding Principles in the Use of Animals in Toxicology” adopted by the Society of Toxicology (USA) in March, 1999. The study was approved by Animal Study Ethics Committee of Wonkwang University.

### 4.8. Animal Experiments Design

*In vivo* study was carried out according to the previous reports with some modifications [15]. Subacute liver injury was induced by CCl_4_ (0.75 mL/kg, p.o., diluted in corn oil) administered every 3 days for 13 days. Vehicle controlled animals were administered corn oil (0.75 mL/kg). Either PF2401-SF (50 mg/kg, p.o., 1 h after CCl_4_ administration in day 13 and 14) or reference drug Gliotoxin (3 mg/kg, i.p., in day 14) were also given. Rats were euthanized by cardiac puncture under ether anesthesia 48 h after the final injection of CCl_4_ administration, and liver tissues were collected for further processing of histological analysis.

### 4.9. Immunohistochemical Staining for α-SMA in Rat Liver

The effect of PF2401-SF on HSCs cells was assessed by immunohistological observation of liver stained with α-SMA muscle as previously reported [36], which is a typical marker of activated HSCs. The portion of removed liver was rapidly fixed by immersion in 10% neutralized formalin (pH 7.4) for 24 h and then paraffin embedded. To detect α-SMA in the liver, tissue sections were deparaffinized, and immunohistochemical staining was performed by the strepavidin-biotin-peroxidase complex method using LSAB^®^ 2 kit (DAKO Co., Carpinteria, CA, USA) and monoclonal anti-α-SMA as a primary antibody.

### 4.10. TUNEL Assay

TUNEL staining was performed according to previous report [8]. For TdT mediated UTP Nick-End Labelling (TUNEL) staining, cells were fixed with 4% paraformaldehyde, and TUNEL Staining was performed using a commercial kit (Dead End^®^ Fluorometric TUNEL system, Promega Corp., Aadison, WI, USA). Cells were then incubated with propidium iodide (PI, 1 µg/mL) for 15 min to visualize all nuclei. Specimen were observed and photographed under a confocal laser microscope (LSM510, Carl Zeiss Inc., Thornwood, NJ, USA).

### 4.11. Statistical Analysis

Results are expressed as mean ± S.D. of independent experiments. Statistical significance of difference between the groups was determined by a Student’s t-test analysis of variation. Values of *p* < 0.05 were considered significant.

## 5. Conclusions

In conclusion, PF2401-SF, the standardized fraction from *S. miltiorrhiza* induces apoptosis of t-HSC/Cl-6 cells through a caspase-mediated pathway *in vitro*. The apoptosis induction of activated HSCs *in vivo* was demonstrated by reduction of α-SMA-positive cells and an increase number of TUNEL-positive cells. Our data provide evidence that PF2401-SF induces apoptosis of activated HSCs and may be a potential herbal medicine for the treatment of liver fibrosis.
